# Supplementary material for: Patterns of social-affective responses to trauma exposure and their relation to psychopathology
Source: PLoS One. 2024 Mar 5;19(3):e0289664. doi: 10.1371/journal.pone.0289664 (PMC10914253; doi:10.1371/journal.pone.0289664)
Supplement: S1 Table — (DOCX) [file pone.0289664.s001.docx]

**S1 Table. Comparison of participants included versus excluded due to missing data**

| Characteristics | Included (n=1321) | Excluded (n=211) | P value |
| --- | --- | --- | --- |
| Depressive symptoms (*M* (*SD*)) | 2.3 (2.7) | 2.0 (2.7) | .108 |
| Anxiety symptoms (*M* (*SD*)) | 3.1 (2.8) | 2.7 (2.6) | .066 |
| PTSD n (%) | 54 (4.1%) | 4 (1.9%) | .131 |
| DD n (%) | 53 (4.0%) | 2 (1.0%) | .042 |
| AUD n (%) | 66 (5.0%) | 12 (5.7%) | .627 |

*Note.* PTSD = Posttraumatic Stress Disorder. AUD = Alcohol Use Disorder. DD = Depressive disorder. *M* = Mean value. *SD* = Standard deviation.
