# Supplementary material for: Patterns of social-affective responses to trauma exposure and their relation to psychopathology
Source: PLoS One. 2024 Mar 5;19(3):e0289664. doi: 10.1371/journal.pone.0289664 (PMC10914253; doi:10.1371/journal.pone.0289664)
Supplement: S2 Table — (DOCX) [file pone.0289664.s002.docx]

**S2 Table. Distribution of items measuring guilt, revenge, shame and social alienation**

|  | “The way I thought/felt and behaved during the event is unforgivable” | “I want to punish the people who did this to me” | “I embarrassed myself during the event” | “If people knew what happened, they would look down on me” | “I will never be able to be close to other people again” | “Other people do not understand me” |
| --- | --- | --- | --- | --- | --- | --- |
|  | *n* | *n* (%) | *n* (%) | *n* (%) | *n* (%) | *n* (%) |
| Strongly disagree | 1105 (83.7%) | 1028 (77.8%) | 1156 (87.5%) | 1104 (83.6%) | 1148 (86.9%) | 952 (72.1%) |
| Rather disagree | 67 (5.1%) | 62 (4.7%) | 54 (4.1%) | 80 (6.1%) | 70 (5.3%) | 138 (10.5%) |
| Neutral | 133 (10.1%) | 160 (12.1%) | 103 (7.8%) | 125 (9.5%) | 90 (6.8%) | 154 (11.7%) |
| Rather agree | 15 (1.1%) | 43 (3.3%) | 5 (0.4%) | 9 (0.7%) | 12 (0.9%) | 64 (4.8%) |
| Strongly agree | 1 (0.1%) | 28 (2.1%) | 3 (0.2%) | 3 (0.2%) | 1 (0.1%) | 13 (1.0%) |
| Total *N* | 1321 | 1321 | 1321 | 1321 | 1321 | 1321 |
